# Efficacy and safety of traditional Chinese medicine in the treatment of chronic pulmonary diseases: a systematic review and meta-analysis

**DOI:** 10.3389/fmed.2025.1512729

**Published:** 2025-05-30

**Authors:** Shan An, Hong-Yan Cai

**Affiliations:** ^1^College of Traditional Chinese Medicine, Changchun University of Chinese Medicine, Changchun, China; ^2^Pulmonology Department, The First Clinical Hospital of Jilin Academy of Traditional Chinese Medicine, Changchun, China

**Keywords:** chronic obstructive pulmonary disease (COPD), traditional Chinese medicine, injections, meta-analysis, systematic review

## Abstract

**Background:**

To assess the efficacy and safety of various TCMIs treatments for COPD using network meta-analysis, providing evidence and guidance for clinical practice.

**Methods:**

We will search 7 databases for randomized controlled trials of TCMI for the COPD, including PubMed, the Cochrane Library, EMbase, China National Knowledge Infrastructure, China Biological Medicine, Chinese Scientific Journals Database, and Wan-fang databases, from the date of the establishment of each database to October 31, 2021. The network meta-analysis will be implemented through Aggregate Data Drug Information System 1.16.8 and Stata13.0 software. Pulmonary function included forced expiratory volume in 1 s (FEV1), forced vital capacity (FVC), and FEV1/FVC will be the primary outcomes, FEV1 as a percentage of the estimated value, maximal voluntary ventilation (MVV), MVV as a percentage of the estimated value, 6 min walking distance, The St.

**Results:**

A total of seven appropriate studies were identified, encompassing 490 patients. The quality of the literature was satisfactory, with no significant publication bias detected. The effectiveness rate for patients receiving a combination of TCM and Western Medicine (WM) compared to those on WM alone was evaluated as an odds ratio (OR), with statistical testing yielding *Z* = 6.09. For PO2 levels post-treatment, the mean difference (MD) was reported as 5.92.

**Conclusion:**

The results of this study will evaluate the efficacy and safety of TCMI in the treatment of COPD, and provide decision-making references for future clinical and scientific research.

**Systematic review registration:**

https://www.crd.york.ac.uk/PROSPERO/view/CRD420251047046, identifier CRD420251047046.

## Highlights

Traditional Chinese medicine is safe in the treatment of chronic obstructive pulmonary disease (COPD) complicated with respiratory failure (RF).

• This approach appears to improve treatment effectiveness without causing serious adverse effects,
• This approach indicates a good safety profile for patients receiving this treatment.

## 1 Introduction

Chronic obstructive pulmonary disease (COPD) is a prevalent and diverse condition marked by persistent airway inflammation and progressive airflow limitation due to bronchial obstruction (1). The development and progression of COPD is significantly linked to an imbalance between proteases and anti-proteases, chronic inflammation, oxidative stress, and excessive mucus production. In 2005, COPD was responsible for over 3 million fatalities, ranking as the third leading cause of death globally and accounting for 6% of total deaths worldwide (2). It remains a major contributor to chronic illness and mortality, significantly impairing patient quality of life, with persistent symptoms such as cough and sputum production affecting about 30% of patients (3, 4). Currently, treatment strategies primarily focus on symptom alleviation, reduction of exacerbations, improvement of quality of life, and enhancement of exercise tolerance (5, 6).

Traditional Chinese medicine (TCM) has attracted growing attention due to its therapeutic potential in managing COPD symptoms. Accumulating evidence suggests that integrating TCM with conventional Western medicine can yield better therapeutic outcomes (7, 8). However, there remains uncertainty regarding the strength of evidence supporting the efficacy and safety of specific commercial TCM injections for COPD. The current systematic review specifically focuses on traditional Chinese medicine injections (TCMIs) including Tanreqing injection (TRQI), Danhong injection (DHI), Xuebiqing injection (XBQI), Shenmai injection (SMI), and Huangqi injection (HQI) which were chosen based on three criteria: 1) widespread clinical use in treating COPD, 2) presence of preliminary clinical evidence supporting their therapeutic effects, and 3) well-documented pharmacological actions related to COPD pathology.

These TCM injections contain distinct bioactive components, which exert therapeutic effects mainly through anti-inflammatory, antioxidative, and immunomodulatory mechanisms. For instance, TRQI includes baicalin, chlorogenic acid, and honeysuckle extracts, known for reducing inflammatory cytokines, such as tumor necrosis factor-α (TNF-α), IL-1β, interleukin-6 (IL-6), and IL-8, contributing to a reduction in airway inflammation (9, 10). XBQI is effective in modulating SP-D and CCL18 levels in COPD patients, thereby alleviating their clinical symptoms. DHI contains salvianolic acids and tanshinones, demonstrating notable antioxidative and circulation-enhancing effects. SMI includes ginsenosides and ophiopogonins, recognized for their beneficial role in improving respiratory function and physical condition. HQI primarily comprises astragaloside IV, displaying potent immunomodulatory and anti-inflammatory properties. These injections have shown promising therapeutic effects in various diseases; however, their clinical efficacy is particularly notable in COPD management, where they effectively alleviate symptoms, reduce airway inflammation, and improve patients’ quality of life.

This systematic review employs standard meta-analysis methods to comprehensively synthesize available clinical trial data, providing a robust evidence base for healthcare providers regarding the clinical utility of these TCM injections (11, 12). The results aim to guide evidence-based clinical decision-making, enhance understanding of TCM’s therapeutic potential, and offer reliable references for future clinical and scientific research (13–15).

## 2 Methods

### 2.1 Search strategy

Computer retrieval of published RCTs of TCMI for the COPD is conducted in PubMed, the Cochrane Library (issue 10, 2021), EMbase, China National Knowledge Infrastructure, China Biological Medicine, Chinese Scientific Journals Database, and Wan-fang databases. The time limit of document retrieval is from the establishment of each database to October 31, 2021. The language is limited to English and Chinese. In addition, inclusive literature from the field and references from previous evaluations will be manually retrieved to find other potentially relevant articles. Search terms mainly include: “chronic obstructive pulmonary disease”, “chronic obstructive lung disease”, “COPD”, “traditional Chinese medicine”, “injection”, “Tanreqing”, “Danhong”, “Huangqi”, “Shenmai”, “Reduning”, “Xuebijing”, etc. Taking PubMed as an example, the initial retrieval strategy is shown in Table 1 and will be adjusted according to the specific database.

**TABLE 1:** Included literatures and their general information.

First author	Year	Outcome index	Combined medicine treatment	Control
Huang (16)	2015	Treatment efficiency, PO_2_, PCO_2_	50	50
Zhang (17)	2004	Treatment efficiency	30	30
He (18)	2014	Treatment efficiency, PO_2_, PCO_2_, FEV1%	50	50
Li (19)	2008	Treatment efficiency	28	26
Xie (4)	2005	Treatment efficiency, PO_2_, PCO_2_	52	30
Jiu (5)	2008	Treatment efficiency, PO_2_, PCO_2_, FEV1%	30	30
Chen (6)	2016	PO_2_, PCO_2_, FEV1%	64	64

### 2.2 Selection criteria for studies

All patients met the diagnostic criteria recommended by the Global Initiative for Chronic Obstructive Pulmonary Disease (15). Patients with COPD whose forced expiratory volume in 1 s (FEV1)/forced vital capacity (FVC) after bronchodilator inhalation were less than 70% were defined as COPD, and the condition was stable/acute exacerbation. Patients with other serious complications were not included, and the course and severity of the disease were approximately the same regardless of sex, age, nationality, or educational background.

### 2.3 Data extraction and risk of bias assessment

Data extraction was carried out meticulously to gather crucial information such as authorship, publication year, geographic location, study design, sample demographics, treatment protocols, and clinical and mycological outcomes. Two reviewers independently performed the data extraction, and any disagreements were resolved through discussion. After independent extraction, the two reviewers compared results and cross-checked their data extraction forms. Any discrepancies or disagreements were thoroughly discussed, referring to original texts until consensus was reached. When necessary, a third independent reviewer was consulted to resolve the remaining discrepancies.

Risk of bias in each included study was assessed by two independent reviewers using the Cochrane Collaboration Risk of Bias Tool, covering aspects such as random sequence generation, allocation concealment, blinding of participants and personnel, blinding of outcome assessment, incomplete outcome data, selective reporting, and other potential biases. Each domain was evaluated and categorized as “low risk,” “high risk,” or “unclear risk.” Any disagreements during this process were resolved through discussion and consensus, with involvement of a third reviewer when necessary.

### 2.4 Statistical analysis

Statistical analysis was performed using Review Manager software (RevMan 5.4, Cochrane Collaboration). The therapeutic efficacy between the combined TCM and Western Medicine (WM) treatment group versus the WM alone group was assessed using odds ratios (ORs) and mean differences (MDs) with 95% confidence intervals (95% CI). Heterogeneity among studies was evaluated using the Chi-square test and quantified by the I^2^ statistic. A fixed-effects model (FEM) was applied if heterogeneity was low (I^2^ < 50% and *P* > 0.1), otherwise, a random-effects model (REM) was used. Sensitivity analysis and subgroup analysis were conducted when significant heterogeneity was detected to explore its sources.

## 3 Results

### 3.1 Literature search and selection

**FIGURE 1:**
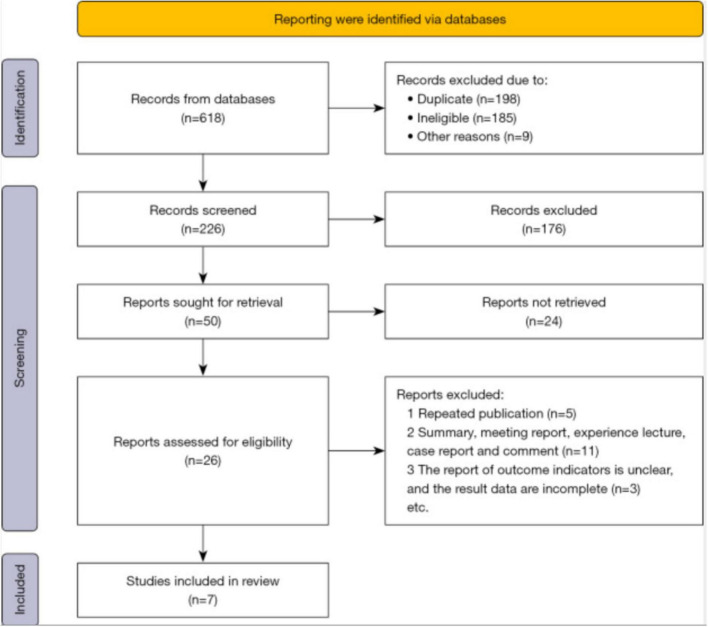
PRISMA flow diagram of study selection process.

Initially, a total of 618 records were collected, and after removing duplicates, 226 abstracts relevant to the specified topic were identified. Two researchers reviewed the titles and abstracts of these articles, resulting in 26 studies that met the inclusion criteria during the preliminary screening phase. Further examination of the full texts led to the exclusion of non-randomized studies, those with repeat data, and literature that was not accessible and 7 articles (Figure 1) meeting the requirements (Table 1) were obtained and included in this study (4–6, 16–19).

### 3.2 Risk of bias assessment

**FIGURE 2:**
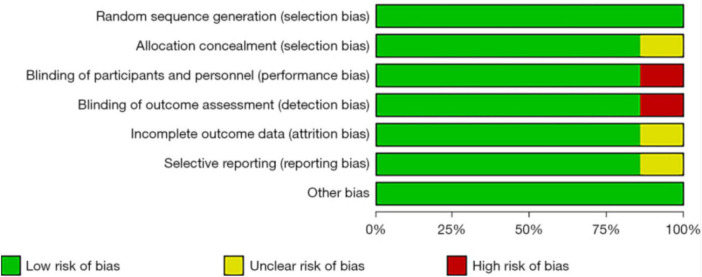
Risk of bias assessment for included studies.

The bias risk was assessed using Cochrane Systematic Review Manual (Figure 2). All seven studies showed low risk regarding random sequence generation, incomplete outcome data, and selective outcome reporting. Overall, the risk of bias in included studies was relatively low, supporting the reliability of the analysis.

### 3.3 Therapeutic efficiency of combined TCM and WM

**FIGURE 3:**
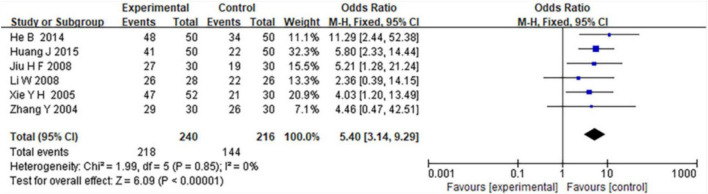
Forest plot comparing therapeutic efficacy of combined TCM and WM versus WM alone in COPD patients with RF.

Six included studies provided detailed evaluations of the therapeutic efficiency of combined TCM and WM treatment versus WM alone in patients with COPD complicated by RF. The meta-analysis indicated that combined TCM and WM treatment resulted in significantly greater clinical efficacy compared with WM alone (MD = 5.40, 95% CI: 3.14–9.29; *Z* = 6.09, *P* < 0.00001), (Figure 3). Additionally, no significant heterogeneity was detected among the included studies (I^2^ = 0%, *P* = 0.85).

### 3.4 Improvement in oxygen partial pressure (PO2)

**FIGURE 4:**
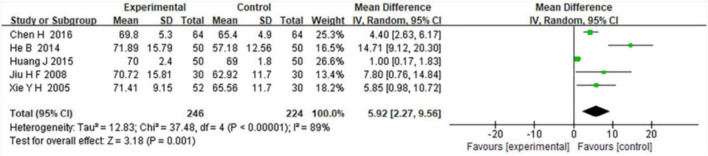
Forest plot evaluating PO2 improvement in COPD patients with RF treated by combined TCM and WM versus WM alone.

Five included studies evaluated changes in oxygen partial pressure (PO2) after treatment. Meta-analysis showed that combined TCM and WM significantly increased PO2 compared to WM alone (MD = 5.92, 95% CI: 2.27–9.56; *Z* = 3.18, *P* = 0.001), although substantial heterogeneity was present (I^2^ = 89%, *P* < 0.00001) (Figure 4).

### 3.5 Changes in partial pressure of carbon dioxide (PCO2)

**FIGURE 5:**
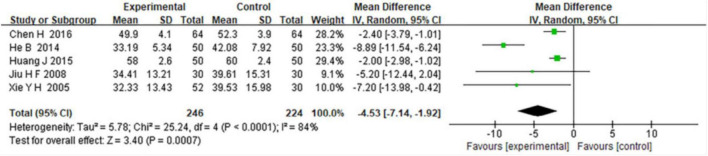
Forest plot comparing the effects of combined TCM and WM versus WM alone on reducing PCO2 in COPD patients with RF.

Five included studies assessed post-treatment partial pressure of carbon dioxide (PCO2) levels in patients receiving combined TCM and WM versus WM alone. Meta-analysis indicated significant improvement (reduction) in PCO2 in the combined TCM and WM group compared with WM alone (MD = −4.53, 95% CI: −7.14 to −1.92; *Z* = 3.40, *P* = 0.0007), although notable heterogeneity was observed (I^2^ = 84%, *P* = 0.0001) (Figure 5).

## 4 Discussion

The results of this systematic review and meta-analysis indicate that the combination of TCMIs with conventional Western medicine demonstrates significant clinical advantages in the treatment of patients with COPD complicated by respiratory failure. Notably, improvements were observed in arterial oxygen partial pressure (PO2), reductions in carbon dioxide partial pressure (PCO2), and higher overall treatment efficacy, suggesting that TCM may play a beneficial role in alleviating respiratory dysfunction and enhancing pulmonary outcomes.

From a pharmacological perspective, frequently used TCMIs such as Tanreqing, Danhong, Huangqi, and Shenmai contain bioactive compounds with anti-inflammatory, antioxidant, and immunomodulatory properties (20, 21). These include flavonoids (e.g., baicalin), phenolic acids (e.g., chlorogenic acid), and saponins (e.g., ginsenosides, astragaloside IV). Previous pharmacological studies have demonstrated that these constituents can inhibit the expression of pro-inflammatory cytokines (e.g., TNF-α, IL-1β, IL-6) and adhesion molecules (e.g., sICAM-1), suppress oxidative stress, and reduce airway inflammation (16, 22). This aligns with our findings, in which the combination therapy group showed notable improvement in gas exchange indicators, further supporting the mechanistic plausibility of TCMI efficacy in COPD management. Additionally, the anti-inflammatory cytokine IL-10 is believed to contribute to the efficacy of TCM by suppressing inflammatory responses and promoting immune balance (17, 18). Emerging evidence suggests that TCMIs exert immunoregulatory effects by modulating signaling pathways such as NF-κB and MAPK and by restoring Th17/Treg homeostasis — mechanisms that may underlie the observed improvements in lung function.

In light of systematic analyses conducted within this study framework, findings reveal that phlegm heat injection can lead to improvements in lung function parameters such as PO_2_ levels while decreasing PCO_2_ levels (19). These results suggest significant anti-inflammatory effects attributed to phlegm heat injection therapy. Consequently, this treatment modality appears effective not only in alleviating clinical signs but also in addressing symptoms experienced by patients suffering from COPD alongside respiratory failure. In terms of safety, some included studies reported mild adverse effects such as nausea, dizziness, and chest tightness following TCMI administration. These reactions were generally transient and resolved spontaneously after discontinuation of treatment, suggesting a favorable short-term safety profile. Nonetheless, individual variability in response should be considered in clinical practice, and close monitoring is advised.

Despite the valuable clinical implications of our findings, several limitations should be considered. First, the included trials had relatively small sample sizes (30–64 participants), which may reduce statistical power and lead to imprecise effect estimates. Second, methodological quality varied, with some studies lacking clear descriptions of randomization or blinding, raising potential risks of bias. Third, substantial heterogeneity was observed in PO_2_ and PCO_2_ outcomes (I^2^ = 89% and 84%, respectively), likely due to differences in disease stages, TCMI types and dosages, treatment durations, and outcome measurements. Although a random-effects model was used, residual heterogeneity may still affect result robustness. Finally, treatment durations were short (7–14 days), with no long-term follow-up data available. Thus, the sustained efficacy and long-term safety of TCMIs remain unclear. Future high-quality, large-scale, and long-term trials are warranted to confirm these findings.

## 5 Conclusion

In summary, the findings suggest that TCMIs are effective and safe in the treatment of COPD complicated with RF. The combined therapy improves clinical outcomes without inducing serious adverse effects, indicating a favorable safety profile and therapeutic potential for integrated TCM and Western medicine approaches.

## Data Availability

The raw data supporting the conclusions of this article will be made available by the authors, without undue reservation.
